# Understanding the taxonomic homogenization of road-influenced plant assemblages in the Qionglai mountain range: A functional and phylogenetic perspective

**DOI:** 10.3389/fpls.2022.1086185

**Published:** 2023-01-09

**Authors:** Honglin Li, Peng Luo, Hao Yang, Wenwen Xie, Chuan Luo, Honghong Jia, Yue Cheng, Yu Huang

**Affiliations:** ^1^ CAS Key Laboratory of Mountain Ecological Restoration and Bioresource Utilization & Ecological Restoration and Biodiversity Conservation Key Laboratory of Sichuan Province, Chengdu Institute of Biology, Chinese Academy of Sciences, Chengdu, China; ^2^ College of Life Sciences, University of Chinese Academy of Sciences, Beijing, China

**Keywords:** mountain roads, biotic homogenization, functional traits, evolution history, biodiversity hotspots

## Abstract

As an increasingly prevalent form of human activity, roads drive the taxonomic homogenization of mountain plant assemblages, threatening global biodiversity. However, little is known about how mountain roads impact functional and phylogenetic beta diversity and how these effects are related to taxonomic homogenization. To understand the mechanism of taxonomic homogenization triggered by mountain roads, we used species absence/presence data from 76 plots (2 m*50 m) and values for 12 traits measured on 978 species from the interior and roadside communities in the Qionglai mountain range, one of the temperate regions with the highest plant species richness in the world. We used a structural equation modeling approach (SEM) to consider several surrogates of road disturbance (changes in soil physicochemical properties and the presence or absence of roads) and the causal relationship between three facets of beta diversity (taxonomic beta diversity, TBD; functional beta diversity, FBD and phylogenetic beta diversity, PBD). The results suggest that TBD, FBD and PBD respond inconsistently to mountain roads, despite strong positive correlations between the three facets of plant beta diversity in the study area. Compared with the interior community, the βtotal.tax and βtotal.func of the roadside community decreased by 2.54% and 2.22%, respectively, which were related to the reduction of species and trait richness differences and replacements; however, we did not find the same results when assessing the changes in βtotal.phy, which represents tip-weighted PBD (twPBD). Furthermore, the largest effect of roads on beta diversity was reflected in basal-weighted PBD (bwPBD), which decreased by 9.97%, indicating that those species with fewer extant relatives and longer evolutionary histories are more sensitive to mountain roads. Therefore, it is necessary to take targeted protection measures for ancient species in roadside communities. In addition, we believe that it is still necessary to take measures to prevent the further dispersal of nonnative species, although the presence of non-native species in roadside plots has led to small changes in three facets of beta diversity. There were causal relationships between the three facets of beta diversity, but their intensity and sign different in the SEM of different components of beta diversity (i.e., richness difference and replacement). Our findings suggest that the homogenization of community species composition at the landscape scale arises by a combination of adaptive responses of the functional traits of organisms to environmental consistency (e.g., reduced the differences in soil variables) caused by roads and resorting or reassembly of community clades composition due to environmental filtering. These results contribute to our comprehensive understanding of the impact of mountain roads on plant diversity, which highlights the complex relationship between human pressure and biodiversity loss.

## 1 Introduction

Worldwide, mountain regions are not only rich in biodiversity (Brighenti et al., 2021), but also often socially and economically underdeveloped (Miller et al., 2021). To support and promote social and economic development, the number of roads in pristine wilderness mountain regions is increasing (Laurance and Balmford, 2013) and becoming an increasingly prevalent and influential form of human disturbance in natural ecosystems. Previous studies have demonstrated that road construction often creates a fundamentally changed environment by clearing vegetation and transforming land use (Forman and Alexander, 1998; Saunders et al., 2002). New roadside habitats are then repopulated with disturbance-tolerant plant species that germinate after road construction and/or disperse from various elevations and surrounding areas (Haider et al., 2018; Rashid et al., 2021). With the opening of roads, roadside environmental conditions are affected by passing vehicle traffic, which further affects the species composition of the roadside plant community (Barbosa et al., 2010). Studies on the impact of roads on the species composition of plant communities have already found that mountain roads result in taxonomic homogenization by diminishing topographic barriers to disperse plant species (Arévalo et al., 2010; Alexander et al., 2016; Lembrechts et al., 2017; Haider et al., 2018).

Moreover, the response of each plant species to a particular environment is expected to be nonrandom depending on their different traits (Götzenberger et al., 2012; Rader et al., 2014; Brice et al., 2017; Arnan et al., 2020). That is, environmental changes caused by human activities may be an important environmental filter that excludes many species with unfit traits, and retains/introduces some species with fit traits. In addition, depending on whether there are phylogenetic signals for these traits (Dehling et al., 2014; Hurtado et al., 2019), community composition may also depend on the evolutionary history of species-adapted traits (Webb et al., 2002; Pillar and Duarte, 2010; McFadden et al., 2019). In other words, the patterns of species co-occurrence in road-influenced landscapes depend not only on environmental pressures but also on functional traits and evolutionary history (Li et al., 2018).

Previous studies on the responses of three facets of plant diversity, namely taxonomy, function and phylogenetic diversity, to environmental changes caused by human activities have mainly focused on the alpha level and analyzed the covariation patterns (Biswas and Mallik, 2011; Lososová et al., 2016) and interactions (López-Angulo et al., 2020; Gianasi et al., 2021) of those responses. Studies on this topic at the beta level are still scarce (Swenson et al., 2012; Myers et al., 2015; Padullés Cubino et al., 2020), although biotic homogenization (the reduction of beta diversity) is one of the most prominent forms of the current biodiversity crisis and is assumed to have an important bearing on future trends in biodiversity (Daru et al., 2021; Yang et al., 2021). The simultaneous analysis of the response of different facets of beta diversity to roads and the interaction between different facets of diversity is important for understanding the mechanisms underlying community species coexistence at the landscape scale.

We focused our study on the Qionglai mountain range in Sichuan Province, China, which is the most plant-rich temperate region in the world (Boufford, 2014; Yao et al., 2020) and a vital part of Giant Panda National Park. Anthropogenic activities are limited to a certain extent in this area, which provides a more ideal place to study the effects of road construction and operation on plant diversity. As a linear landscape, the road is a narrow but very long gap in natural ecosystems, creating contrasting environment condition (e.g., microclimatic and soil conditions) compared to adjacent natural ecosystems (Laurance et al., 2009; Kunert et al., 2015). Such an edge, or boundary environment has caused taxonomic homogenization by excluding many plant species from roadsides and attracting the colonization of pioneer species in the Qionglai mountain range (Li et al., 2022a). And it also favored or excluded particular functional traits and lineages in roadside communities, resulting in the differences of the function and phylogenetic composition between roadside and interior plant communities (Li et al., 2022b). This would result in the replacement or loss/gain of functional traits and lineages within the plant community. To help management agencies draw up effective conservation management practices for roads within national park, there is an urgent need to determine the relationship between taxonomic homogenization and the impact of roads on the function and phylogenetic composition of plant communities.

Therefore, our objective in this study was to evaluate whether and how mountain roads act on functional beta diversity (FBD) and phylogenetic beta diversity (PBD). On this basis, we assumed that the environmental changes caused by roads primarily affect FBD and then regulate the changes in taxonomic beta diversity (TBD) and PBD (**Figure 2**). Meanwhile, we hypothesized that there is also a causal relationship between PBD and TBD. Moreover, we also considered the responses of PBD at different evolutionary depths (basal-weighted PBD, hereafter “bwPBD” and tip-weighted PBD, hereafter “twPBD”) to roads in the analysis of PBD. We investigated these points by asking the following questions: (i) Are the effects of mountain roads on functional and phylogenetic beta diversity consistent with the effects of taxonomic beta diversity, that is, do roads also cause functional and phylogenetic homogenization? (ii) What is the relationship between taxonomic, functional and phylogenetic beta diversity in road-driven changes in taxonomic beta diversity? The main drive for this research is to provide theoretical support for finding solutions for the sustainable development of roads within nature reserves and realizing the mutual benefit for people and biodiversity conservation.

## 2 Materials and methods

### 2.1 Study region

**Figure 1:**
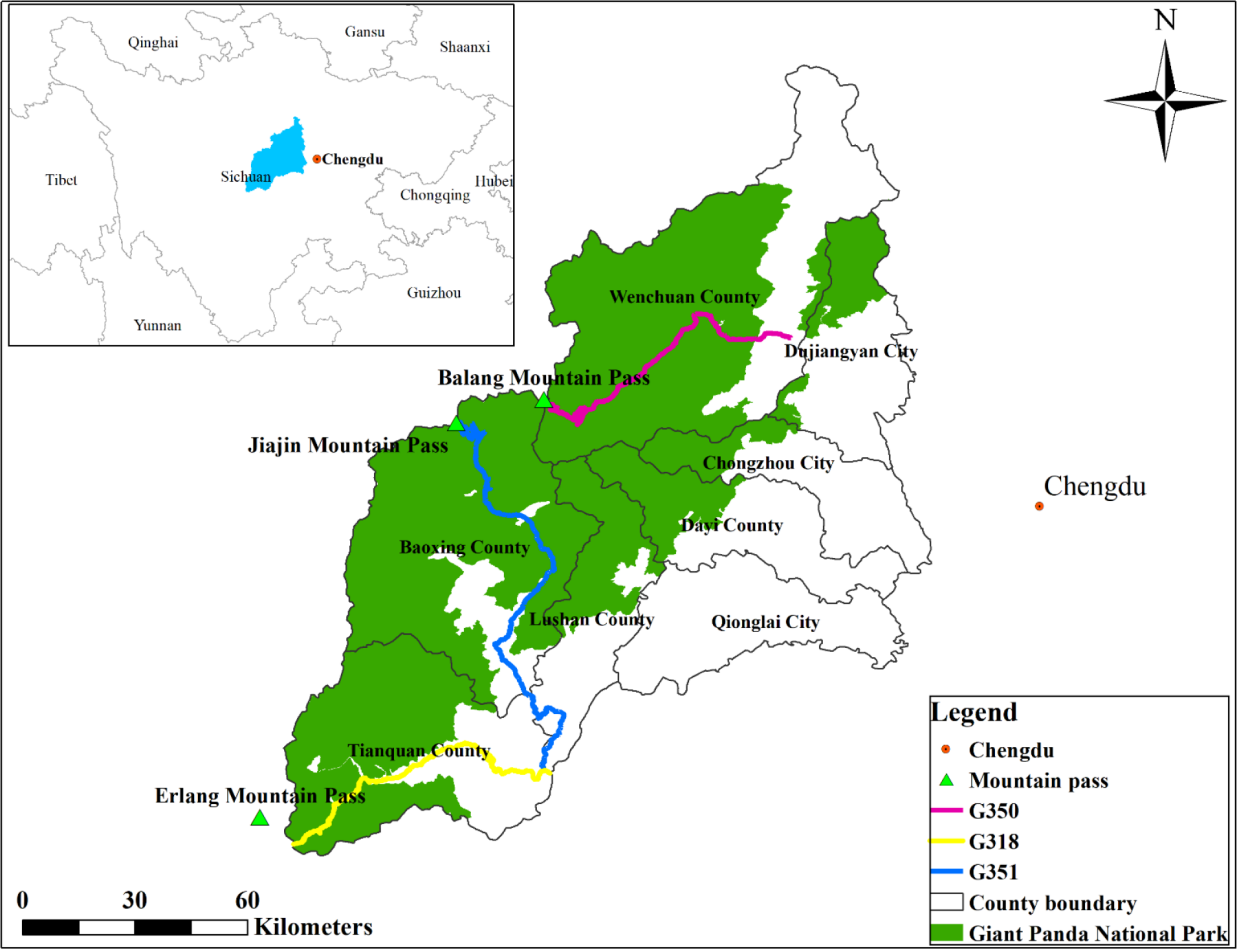
Locations of three major roads in the Qionglai mountain range.

In 2019 and 2020, we conducted a plant community survey along the only 3 major roads which pass through Giant Panda National Park in the Qionglai mountain range (eastern part of the Hengduan Mountains). In this study, we selected segments of the three roads located within Giant Panda National Park (**Figure 1**). These road segments had similar attributes (i.e., concrete pavement, 8.5 meters of road wide and more than 50 years of operation). For more details on the sites, see Li et al. (2022a, 2022b).

### 2.2 Sampling design

We selected 38 sampling sites along the three major roads. According to the elevation range of the selected section of each road, 5, 16 and 17 sampling sites were set at G318, G350 and G351, respectively. To reduce the mutual interference between sampling sites, the sites were separated by approximately 1,000 m. The sampling sites must be located at least 1,000 m from farmland or human settlements. At each site, we inventoried vascular plants in a pair of 2 m × 50 m plots (76 plots). One plot of the paired plots was established parallel to and bordering the cleared road corridor. The other plot was located within the natural plant community, more than 50 m away from the roadside.

### 2.3 Measure/collection of functional traits

We collected data on 12 functional traits for each species (**Table S1**). These traits were relatively uncorrelated with each other (ρ< 0.5) (**Figure S1**). Three quantitative functional traits, specific leaf area (SLA), leaf dry matter content (LDMC), and leaf thickness (Lth), were measured in the laboratory. LDMC and Lth were determined according to the procedures of Pérez-Harguindeguy et al. (2013). Leaf area was measured following Cornelissen et al. (2003). Ten to 30 healthy mature individuals were selected for each species from all plots, and 2 intact and healthy mature leaves were collected from each individual. Half of the leaves were used for the determination of LDMC, and the other half were used for the determination of Lth and leaf area. For the species that have collected enough leaves in the previous sampling, no further collection would be conducted in the subsequent sampling. For the species that have not taken enough samples in the previous sampling, continue to collect in the subsequent sampling. The mean value of each functional trait was taken for subsequent calculation. Data for the other nine traits were obtained from the *Flora of China* (English edition, www.iplant.cn and www.efloras.org) and the TRY database (http://www.try-db.org/) (Kattge et al., 2020).

### 2.4 Soil sample collection and analysis

In each field plot, soil samples were collected from the surface soil (0-20 cm). A total of five soil samples in self-sealing bags and five soil samples in cutting rings were collected from each plot, and the soil parameters were the average of the five samples. Soil samples with cutting rings were weighed and then dried at 105°C to measure bulk density (BK, g/cm^3^) (Sun et al., 2020). The soil samples in self-sealing bags were air-dried, and the dried samples were ground, sieved, and stored at ambient temperature for subsequent analysis of soil chemical properties. Since sampling occurred in the rainy season, the soil moisture content was not determined.

The chemical properties of the soil were analyzed according to Lu (2000). The soil pH value and electrical conductivity salinity (EC, μS/cm) were determined with a pH meter and conductivity meter (soil-to-water ratio, 1:2.5), respectively. The soil organic matter content (SOM, g/kg) was determined by the potassium sulfate dichromate oxidation method. The soil total nitrogen content (TN, g/kg) was measured using the Kjeldahl method according to Bremner and Mulvaney (1982). Total phosphorus (TP, g/kg) and available phosphorus (AP) (mg/kg) were determined by perchloric acid digestion and sodium bicarbonate extraction, respectively, and then by the molybdenum anti-colorimetric method. ${\text{NH}}_{4}^{+}-\text{N}$ (mg/kg) and ${\text{NO}}_{3}^{-}\text{N}$ (mg/kg) were measured using UV-Vis spectrophotometry. Cation exchange capacity (CEC, cmol/kg) was measured using the sodium (Na) saturation method (Rhoades, 1982).

### 2.5 Calculation of three facets of plant beta diversity

#### 2.5.1 Phylogenetic tree

We assembled a phylogenetic tree with all 978 species from the pruned mega-tree GBOTB.extended.tre by Zanne et al. (2014) *via* the options of scenario 3 with V. PhyloMaker package (Jin and Qian, 2019).

#### 2.5.2 Functional dendrogram

FBD and its two components were measured based on 12 functional traits of the species. Information on plant habits was extracted at the species level, and in case of missing information, we used families as the upper limit for classification. Gower’s distance was used to obtain the distances of 12 traits among all species. The functional dendrogram was then built by the UPGMA method (Villéger et al., 2012; Wayman et al., 2021).

We also estimated the phylogenetic signal for 12 functional traits using the Phytools package with the *phylosig* function to confirm the relationships between FBD and PBD (Pagel, 1999).

#### 2.5.3 Metrics of TBD, FBD and PBD

Beta diversity was calculated as a pairwise comparison of species composition across all plots located within the same plot category, namely, interior or roadside plots. Changes in beta diversity provide opportunities for understanding species replacement, changes in species richness, and the effects of other environmental differences (Baselga, 2010; Baselga, 2012; Cardoso et al., 2014). We analyzed the effects of roads on the multiple facets of beta diversity in terms of two components of beta diversity.

We measured TBD, FBD and PBD using the Jaccard dissimilarity index (βtotal) and partitioned βtotal into two distinct components, βrepl and βrich. βtotal and its two components are calculated as follows (Podani and Schmera, 2011):


βtotal=(b+c)/(a+b+c).

βrepl=2∗min(b,c)/(a+b+c).

βrich=|b−c|/a+b+c.

When TBD and its components are calculated, a is the number of species shared by both plant communities, b is the number of species unique to one plant community, and c is the number of species unique to the other community (Podani and Schmera, 2011). When the above formula is used to calculate FBD and its components, shared and unique species are replaced with shared and unique traits from the functional tree, respectively. When PBD and its components are calculated, shared and unique species are in turn replaced by shared and unique lineages in the phylogenetic tree, respectively. The beta diversity was calculated for all plot pairs using the Biodiversity Assessment Tools (BAT) package (Cardoso et al., 2015).

The metric based on dissimilarity is the twPBD metric (Swenson, 2011; Qian et al., 2020). Tip-weighted PBD is focused on the terminals, where it is sensitive to replacement among recently diverged phylogenetic branches within clades (Qian et al., 2021). Basal-weighted PBD is more sensitive to changes at the roots of the phylogenetic dendrogram (Saunders et al., 2002; Swenson, 2011; McFadden et al., 2019). We calculated the mean pairwise distance (MPD) of separated taxa in two communities from the *comdist* function in the picante package to characterize the bwPBD (Kembel et al., 2010). Using both twPBD and bwPBD measures helps to understand where the species affected by roads appear on the phylogenetic tree.

### 2.6 Statistical analyses

For the first question, we used linear mixed effects models to compare TBD, FBD, and PBD in roadside and interior communities, and used mantel analysis to analyze the effects of roads on the correlations between three facets of beta diversity. Regarding our second question, we examined the correlation between TBD, FBD and PBD in roadside and interior communities and tested the direct and indirect effects of road-induced environmental changes on the three facets of beta diversity using SEM.

#### 2.6.1 Linear mixed effect model

As the plots were nested for each site and the sites were nested for each road, we used a linear mixed effects model to analyze the effects of mountain roads on TBD, FBD and PBD and their components, with plot as random factors. First, we calculated the average beta diversity for each plot from all plot pairs within the same plot category (interior or roadside plot) that included the plot and used the average beta diversity per plot in the linear mixed effects model. Second, the linear mixed models were fitted using the lme4 package with the *lmer* function (Bates et al., 2015) in R 4.1.0 (R Core Team, 2021).

The full model _(beta diversity)_ = b1 *plot category + (1|plot).

The influences of roads on soil physicochemical properties also referred to the above equation. In addition, we used a linear mixed effects model to analyze the effects of nonnative species on three facets of beta diversity in roadside communities.

#### 2.6.2 Mantel analysis

We performed simple Mantel tests with 999 permutations to examine the relationships between three facets of beta diversity. The grading of the degree of correlation in correlation analyses was determined according to the study of Qian et al. (2020). To avoid pseudoreplication, we introduced 703 distance data below the diagonal of the distance matrix of each soil variable and the matrix of each beta diversity index into mental analyses and SEM.

#### 2.6.3 Structural equation modeling

**Figure 2:**
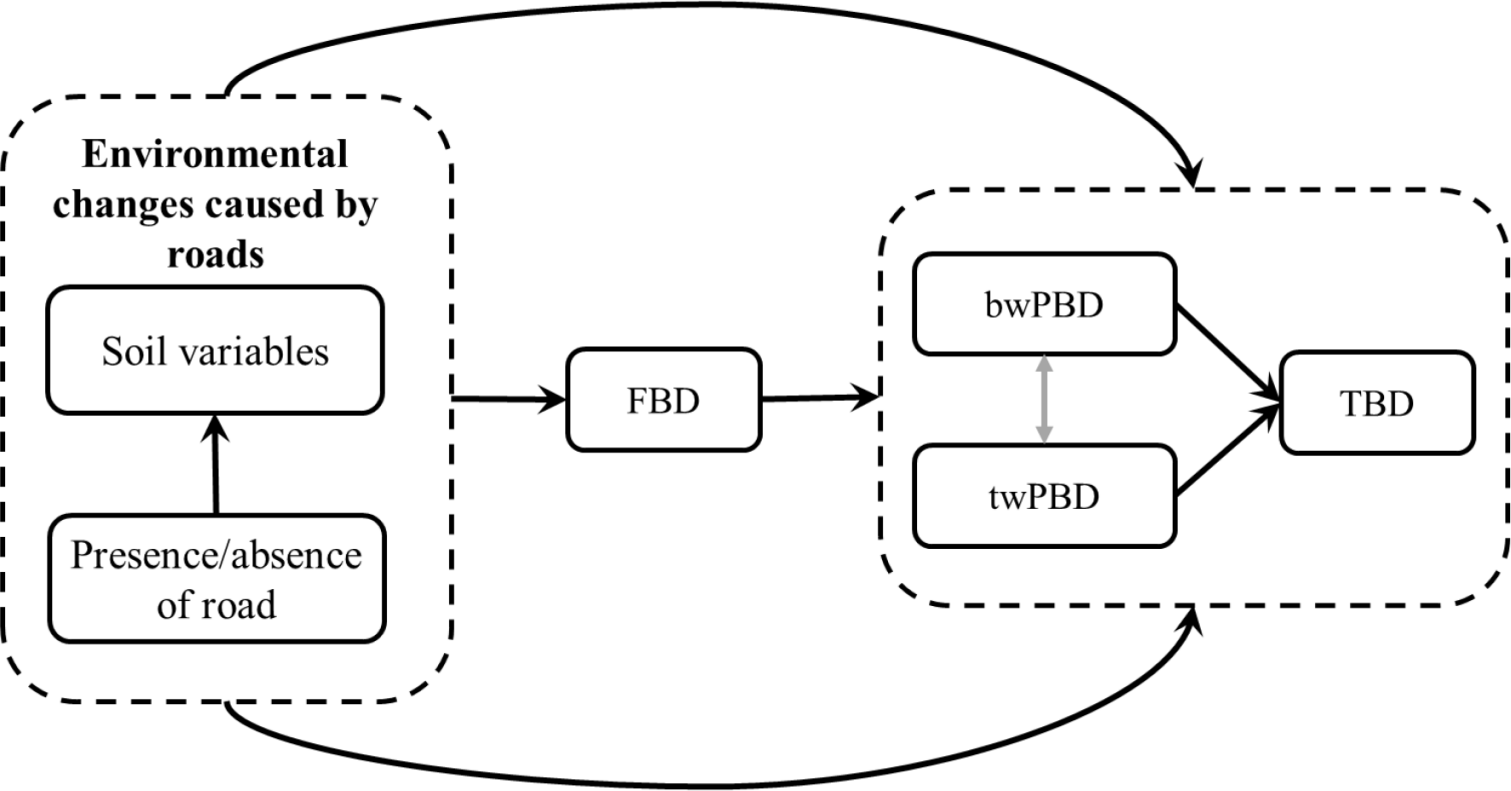
A causal framework, including the presence/absence of mountain roads, change in soil variables and the three facets of beta diversity. Arrows indicate the direction of causality. TBD, taxonomic beta diversity; FBD, functional beta diversity; bwPBD, basal-weighted phylogenetic beta diversity; twPBD, tip-weighted phylogenetic beta diversity.

We used SEM to examine the relationships between environmental changes caused by roads and three facets of beta diversity. Considering that the functional structure of the plant community may have a strong phylogenetic signal (**Table S2**), we hypothesized that shifts in FBD also mediate changes in PBD. The increase of PBD is also expected to be associated with the increased of TBD, since more lineage replacement should mean more species replacement (Losos, 2008). We proposed and tested a causal framework that the multiple facets of plant beta diversity are related, with FBD directly affected by environmental filtering (i.e., the presence/absence of road and change in soil physiochemical properties) caused by roads, while TBD primarily responds to changes in FBD and PBD (**Figure 2**). In addition, the relationship between the twPBD and bwPBD was also considered in the SEM analysis.

To reduce the number of potential exogenous variables, we eliminated those that were strongly correlated (ρ>0.5) with other soil factors (**Figure S2**) and selected the soil variables that were significantly affected by roads according to the results of the linear mixed effects model (**Figure S3**). For all pairwise comparisons of each soil factor distance among plots that were located within the same plot category, we used the Euclidean distance for the R package ecodist with the *distance* function. The distances of the soil factors were put into the SEM for analysis. The SEM was fitted using the function *sem* (R package lavaan) (Rosseel, 2012). The models were tested by maximizing two measures of good fit (>0.9) (Malik et al., 2018) and minimizing two measures of error (<0.05) (Malik et al., 2018). The models with the lower AIC were selected by the Akaike information criterion (AIC), while the model with Δ AIC > 4 was not selected (Borcard et al., 2018). For clarity, nonsignificant positive and negative path coefficients were omitted from the figures and were listed in **Table S3**.

## 3 Results

**Figure 3:**
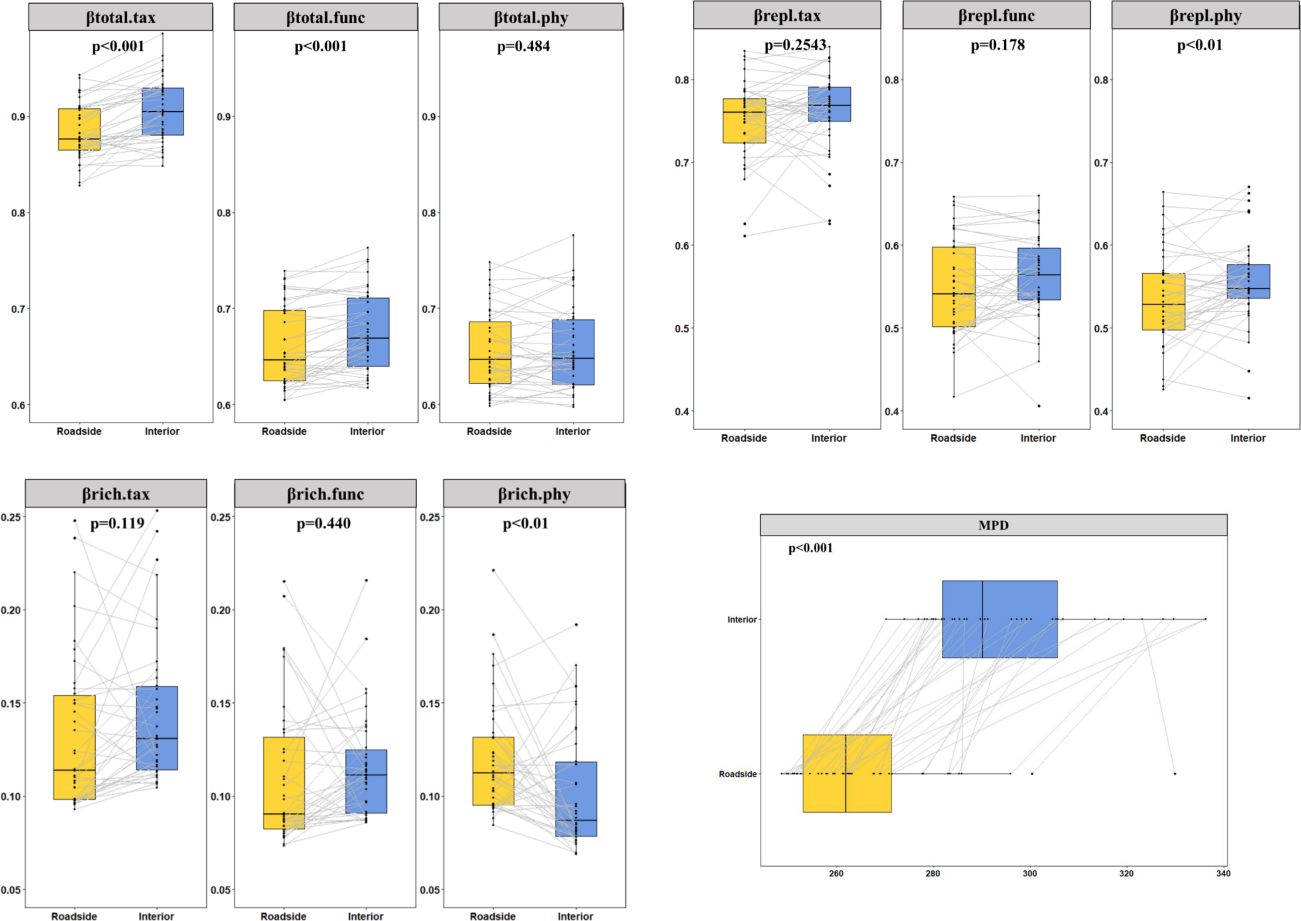
Differences between interior and roadside plots were tested with linear mixed models (n=38). The points represent the mean beta diversity of each plot, and the lines connect the beta diversity of the roadside plot and interior plot at the same sampling point. The p value represents the result of the linear mixed effects model.

A total of 978 species belonging to 395 genera from 113 families were recorded. The results of the linear mixed effect models showed that the roadside environments of its three main roads had higher soil pH values, greater bulk density, lower soil nitrogen and greater carbon stocks (**Figure S3**) than the interior habitats. We further analyzed the impact of mountain roads on TBD, PBD, FBD and their components. Over the whole Qionglai mountain range, road-induced βtotal.tax, βtotal.func, βtotal.phy and bwPBD decreased by 2.54% (p<0.01), 2.22% (p<0.01), 0.46% (p>0.05), and 9.97% (p<0.01), respectively. The two components of βtotal for TBD and FBD did not have a significant difference between the roadside and interior plots, but the βtotal did (**Figure 3**). Compared with the interior plots, bwPBD and the βrepl.phy were significantly lower and the βrich.phy was significantly higher in the roadside plots (**Figure 3**). In addition, nonnative species significantly decreased βrich.tax, βtotal.func, βrich.func and βtotal.phy by 8.80%, 0.30%, 4.61% and 0.26%, respectively, and significantly increased βrepl.tax by 1.80% in roadside communities (**Figure S4**).

**Figure 4:**
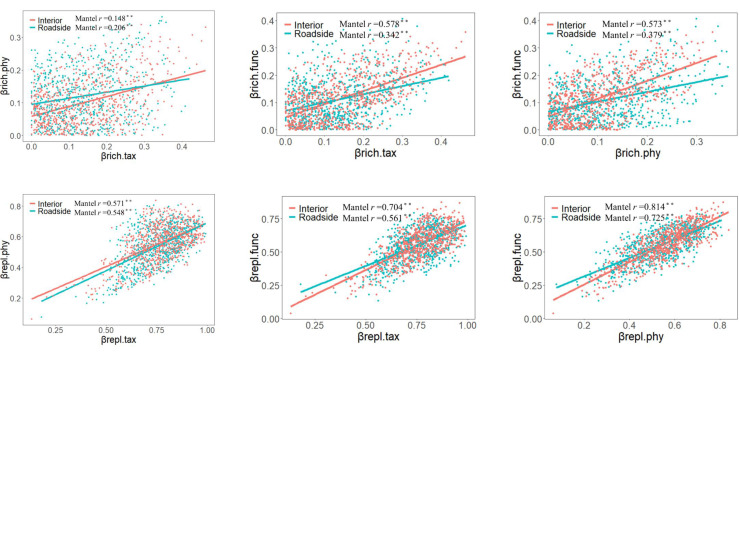
Correlations between taxonomic, phylogenetic, and functional total beta diversity (βtotal.tax, βtotal.phy and βtotal.func), replacement (βrepl.tax, βrepl.phy and βrepl.func), and richness differences (βrich.tax, βrich.phy and βrich.func) (n=703). A Mantel test was applied to assess the significance of the relationship of the Pearson correlation coefficient (r): ** p<0.01.

For all plot categories, the correlations between TBD, FBD and PBDs were significantly positive (such as in interior plots, Pearson’s correlation coefficients: 0.893 for βtotal.tax vs. βtotal.phy, 0.920 for βtotal.tax vs. βtotal.func, and 0.911 for βtotal.phy vs. βtotal.func). Except for βrich for TBD and twPBD, the correlation coefficient for the three facets of beta diversity and their components decreased in roadside communities compared with that in interior communities. These relationships revealed mismatches and congruencies between biodiversity components (**Figure 4**). Moreover, bwPBD was positively correlated with βtotal.phy and βrepl.phy (**Figure S5**).

**Figure 5:**
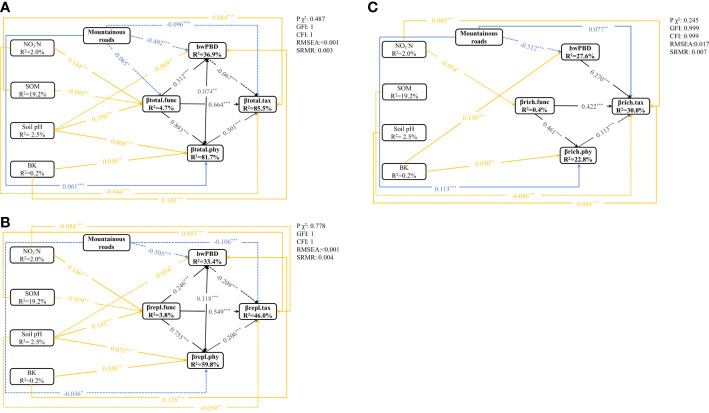
The SEM showed direct and indirect effects of road disturbance, soil variables on βtotal.tax, βtotal.func, βtotal.phy and bwPBD **(A)**, βrepl.tax, βrepl.func, βrepl.phy and bwPBD **(B)**, and βrich.tax, βrich.func, βrich.phy and bwPBD **(C)** (n=703). Boxes indicate measured variables and arrows indicate relationships among variables. Double-headed arrows and single-headed arrows represent a correlative relationship and a causal relationship, respectively. The relationships between the three facets of beta diversity are shown as black lines, the impact of roads on beta diversity is shown as blue lines, and the yellow lines represent the impact of changes in soil physicochemical properties on beta diversity. Solid and broken arrows represent positive relationships and negative effects, respectively. Significance levels were as follows: *P < 0.05, **P < 0.01 and ***P < 0.001.

To reduce model complexity, the relationships between mountain roads and changes in soil variables were plotted separately (**Figure S6**). The SEM for βtotal, βrepl and βrich was guaranteed by a nonsignificant GFI>0.9, CFI>0.9, SRMR<0.05, and RMSEA<0.05 (**Figures 5A–C**). SEM analysis showed that the explained variance reached 85.5% for βtotal.tax, 81.7% for βtotal.phy and 36.9% for bwPBD (**Figure 5A**). In the βrepl model, 33.4%, 59.8% and 46.0% of the variation in bwPBD, twPBD and TBD, respectively, was explained by the investigated variables (**Figure 5B**). In the βrich model, 27.6%, 22.8% and 30.0% of the variation in bwPBD, twPBD and TBD, respectively, was explained by the investigated variables (**Figure 5C**).

**Figure 6:**
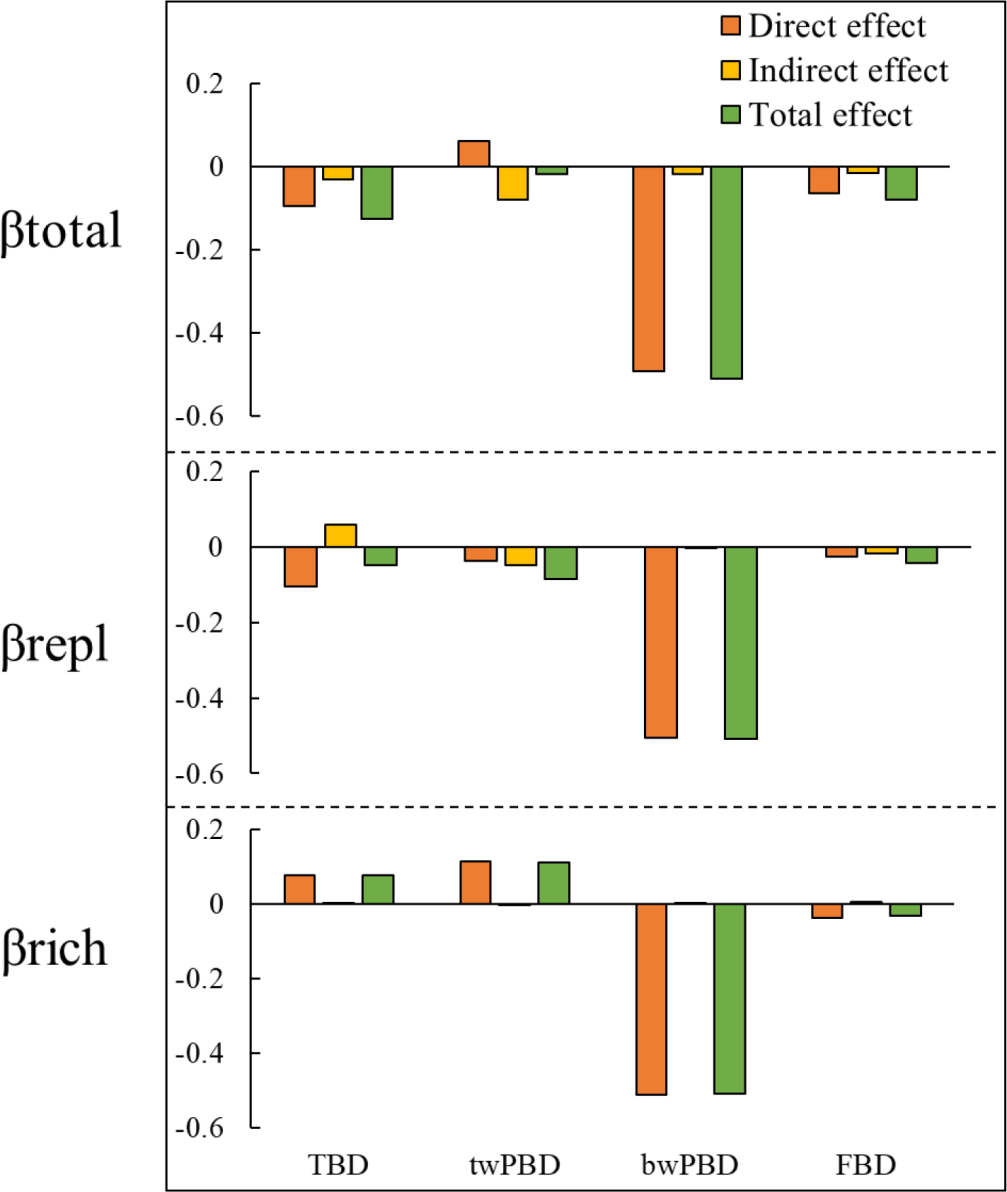
Synthesis of the direct, indirect and total effects of mountain roads on βtotal and its two components. TBD, taxonomic beta diversity; FBD, functional beta diversity; bwPBD, basal-weighted beta diversity; twPBD, tip-weighted phylogenetic beta diversity.

In addition to indirectly affecting TBD through changes in FBD and twPBD, mountain roads also had direct effects on TBD (**Figure 5**). The magnitude of indirect and direct effects varied in the different indicators and two components of beta diversity. While the direct effects of the presence/absence of roads on FBD and TBD were greater, the indirect effects of the presence/absence of roads on twPBD were greater (**Figure 6**). The indirect effects were comparatively stronger in the soil variables (**Table S3**). The presence of roads was associated with lower βtotal.tax, βrepl.phy and βrepl.tax values. However, the presence of roads increased the βrich.tax, βtotal.phy and βrich.phy values (**Figure 5**). In the βrepl and βrich models, the presence/absence of roads had only indirect effects on FBD (**Figure 5B**).

Moreover, there were not always positive relationships among the multiple facets of plant beta diversity (**Figure 5**, **Table S3**), such as the relationship between bwPBD and βtotal.tax and the relationship between bwPBD and βrepl.tax. As most plant functional traits had strong phylogenetic signals (**Table S2**), we also found a significant effect of FBD on twPBD variation in all models (**Figure 5**, **Table S3**). Among the impacts of mountain roads on the three facets of beta diversity in the study area, the impact on bwPBD was the largest, reaching a moderate level (>0.5) (**Figure 6**, **Table S3**).

## 4 Discussion

### 4.1 Roads result in taxonomic homogenization accompanied by functional and phylogenetic homogenization

We found positive correlations between TBD, FBD and PBDs, and the correlation coefficients were reduced by the presence of road, except for bwPBD. Similar to our results, Biswas & Mallik (2011) found positive relationships between the multiple facets of diversity, but the slopes of the relationship varied with disturbance intensity. However, we also found that the correlation coefficients between bwPBD and other facets of beta diversity were increased by the presence of roads. The reason may be that there were more closely related species in the community undisturbed by the road, which resulted in the redundancy of community phylogenetic branches, and the correlation coefficient between bwPBD and TBD was low. When the mountain roads eliminated more closely related species that were relatively deeper within the phylogenetic tree, the bwPBD was more likely to change as species composition changes. As a result, the correlation coefficient between the two facets of beta diversity would increase.

The positive relationship between the three facets of beta diversity suggests that changes in the three metrics do not occur independently. Interestingly, when analyzing twPBD, we found no evidence for a significant change in twPBD in roadside plots, despite conspicuous declines in TBD and FBD. This result is consistent with studies examining changes in phylogenetic diversity following anthropogenic disturbance in the Canadian boreal forest region (Zhang et al., 2014), tropical forests (Arroyo-Rodríguez et al., 2012), and alpine meadows (Yang et al., 2012). However, compared to the above studies, by partitioning twPBD, our results revealed that the significantly increased lineage richness difference caused by the introduction of generalist or nonnative species that are not closely related to native species masked the significant reduction in lineage replacement among roadside plots. The result stresses the need for understanding the impact of environmental filtering on community composition across different ecological processes.

Further partitioning the βtotal of TBD and FBD into their respective βrepl and βrich components also revealed that the homogenization of both taxonomic and functional diversity was driven by a joint reduction in βrepl and βrich, neither of which coincided with the process of change in tip-weighted PBD. Meanwhile, across the Qionglai Mountains, the degrees of taxonomic and functional homogenization in roadside plant communities were 2.54% and 2.22%, respectively. This result is consistent with the patterns already observed in assemblages of natural vegetation (Kearsley et al., 2019; Stotz et al., 2021), suggesting that almost all species have a unique and important functional trait for the natural assemblages of this most botanically rich temperate area. The different responses of the multiple facets of beta diversity and their components to mountain roads challenge the use of any one diversity component as a good surrogate for others.

Previous studies have found 32 nonnative species in the study area (Li et al., 2022a). More nonnative species were found in roadside communities at low elevations, and few or no nonnative species were found in roadside communities at high elevations. In this study, we further explored the effects of nonnative species presence in roadside plots on beta diversity. The results showed that nonnative species reduced the dissimilarity of functional trait composition by reducing differences in trait richness. One possible explanation is that the nonnative species that spread along the road brought functional traits to the community at low elevations that existing species did not. Their entry into low-elevation roadside communities increased the richness of community trait composition and made up for the difference in trait richness between low and high elevations. Since their entry did not result in a significant increase in trait replacement, we speculate that none or only a few of their functional traits differ from those of existing species at high elevations.

In addition, we also found that the dispersal of nonnative species reduced the difference in the composition of recently diverged phylogenetic branches (twPBD) between low and high elevations roadside plant communities. In other words, non-native species may be closely related to some species with recently diverged phylogenetic branches at high elevations. Darwin’s preadaptation hypothesis suggests that the close relatedness between nonnative species and native species of recipient communities can promote nonnative species to adapt well to that community (Ricciardi and Mottiar, 2006), because close relatives share similar functional traits and may favor similar environmental conditions (Li et al., 2015). Based on Darwin’s preadaptation hypothesis, nonnative species in this study area have functional traits to adapt to high elevation environment. Therefore, we believe that an comprehensive environmental management plan for nonnative species in the study area remains necessary in the context of climate change.

### 4.2 Roads alter the FBD and PBD, which in turn leads to taxonomic homogenization

Species traits can offer clues and insights regarding where a plant grows and where it does not (Reich, 2014). From our previous results, ongoing and historical environmental filtering following road construction in the study area narrowed the range of functional traits of species in the montane plant communities, leading to a reduction in functional alpha and beta diversity at the landscape scale (Li et al., 2022b). Our hypothetical framework for the causal relationships between plant beta diversity and changes in environmental conditions caused by roads was confirmed, but the relationships between different indicators in models with different components of beta diversity varied widely (**Figures 5A–C**).

First, we observed indirect effects mediated by changes in the composition of functional traits in plant communities. On the one hand, there were strong positive causal relations between FBD and TBD, as well as between FBD and PBD, indicating that there was phylogenetic conservatism in functional traits (Webb et al., 2002; Swenson & Enquist, 2007) and functional complementarity (Petchey & Gaston, 2002; Stotz et al., 2021) among species in the community. On the other hand, the direct effects of mountain roads on βtotal.phy and βrepl.phy were smaller than the indirect positive effect through FBD. The findings remind us that focusing solely on the direct effects of environmental change may ignore the potential importance of indirect effects, ultimately leading to an incomplete estimation of the effects of anthropogenic activities on natural ecosystems. In conclusion, FBD not only responds earlier and more strongly to environmental changes than other facets of beta diversity, but also predicts changes in other facets of beta diversity.

Second, we also observed a significant causal relation between PBDs and TBD. To the best of our knowledge, causal relationships between phylogenetic and taxonomic diversity in vascular plants have not been found at the alpha level (Hurtado et al., 2019; López-Angulo et al., 2020). The possible reason is that beta diversity, which explicitly considers the species identity of community composition compared to alpha diversity, provides a more sensitive indicator of biotic changes caused by human disturbance (Whittaker et al., 2001). Regardless, the result suggests that environmental changes possibly select species of particular clades from the regional lineage pool, with species retention or exclusion according to the adaptive potential to particular environmental constraints. Altogether, the variation in phylogenetic components of the community at different evolutionary depths better reflects how environmental stress drives plant community assembly.

Moreover, significant causal links varied among different components of beta diversity with shifts from positive to negative in one compared to other components (e.g., mountain roads vs. twPBD in both βrepl and βrich models), which suggests the importance of partitioning beta diversity. Finally, the significant direct effects of roads on TBD and PBDs suggested that soil variables are not the unique surrogate for road effects, nor is FBD the only predictor for the remaining facets of beta diversity. The former was easy to understand, and the latter could be explained by some other relevant traits not included in our study as well as by some stochastic variation unrelated to the functional traits and evolutionary history of the species (Hurtado et al., 2019; López-Angulo et al., 2020).

### 4.3 The role of soil variables

The soil acidity of the adjacent natural ecosystem (pH=6.36) was significantly higher than that of the roadside habitat (pH=7.01), indicating that the roadside habitat changed from acidic soil to neutral or even alkaline soil due to road disturbance. The higher soil pH value in roadside habitats has been attributed to calcareous road dust deposition and leachate (Johnston and Johnston, 2004). In addition, roadside habitats had higher soil bulk density values due to roadside compaction after mountain road construction. Changes in soil pH value and bulk density can affect the use of nitrogen, phosphorus and other minor cations by plants (Auerbach et al., 1997; Johnston and Johnston, 2004), so they are determining factors in the composition of roadside plants (Arenas et al., 2017). These results are consistent with the results found here, with changes in soil pH value, soil bulk density, soil organ matter and NO3^–^N always having stronger effects on the multiple facets of beta diversity than other variables.

As predicted by the environmental filtering hypothesis, any change in abiotic factors in one direction is an environmental filter, resulting in the reduction of differences in trait composition between communities (Grime, 2006; Concostrina-Zubiri et al., 2022). This hypothesis is supported here. The mountain road made the soil environment of roadside habitats neutral, compact and high nutrient content, and this homogeneity of soil conditions reduced the FBD in the study area. Interestingly, we found that in addition to the indirect effects of soil variables on TBD and PBDs (i.e., through changes in FBD), the direct effects of these soil variables were also detected, suggesting a need to simultaneously analyze the response of multiple facets of beta diversity to human activities in the absence of data on all functional traits.

### 4.4 Species located at the relatively deeper nodes in the phylogenetic tree are more sensitive to mountain roads

Two processes are responsible for shifts in phylogenetic diversity: the loss of species and the increase in species relatedness (Frishkoff et al., 2014). In the present study, both processes played a role in the impact of roads on PBDs, as roads led to the loss of native perennial species and the colonization of generalists. Our results showed that the bwPBD declined more than the twPBD. That is, the species that host them are not evenly distributed among nodes at different depths on the phylogenetic tree.

The result indicates that there is a greater decrease among clades relative to within clades, which we infer to mean that environmental filtering in roadside communities tends to exclude all species in various older clades but only a particular species in other, younger clades. One possible reason is that the generalists or nonnative species that spread through mountain roads are typically more closely related to shallow nodes than deep nodes, and their introduction can complement the loss of shallow nodes but not the loss of deep nodes. Old-growth flora in edge-affected habitats were impoverished, as found by Tabarelli et al. (2008); Lôbo et al. (2011) and Tabarelli et al. (2010).

Based on studies of birds (Gaston and Blackburn, 1997; Lockwood et al., 2000), mammals (Johnson et al., 2002; Verde Arregoitia et al., 2013) and plants (Vamosi and Wilson, 2008; Dinnage et al., 2020), there have been many speculations about why species with an earlier evolutionary history are more susceptible to anthropogenic influences. In response to the results of this study, we speculate that the possible reason is that old lineages may happen to not have the traits to adapt to human-caused environmental change, and the current environmental changes are faster than the rate at which plants have evolved fit traits, preventing them from appearing in human-dominated landscapes.

## 5 Conclusions and implications for management and conservation

Our results showed that the taxonomic, functional and phylogenetic beta diversity did not always respond similarly to mountain roads, although they were closely related, confirming that taxonomic beta diversity was a poor surrogate for functional and phylogenetic beta diversity. Moreover, as hypothesized, shifts in trait and lineage composition mediated the effects of mountain roads on the species composition of the montane plant community. The relative importance and effect direction of these drivers differed in different components of beta diversity (i.e., richness difference and replacement). This observation highlights that the species sensitive to anthropogenic activities are not randomly distributed among taxa, not only because of functional traits but also because of their evolutionary age. Based on the aforementioned results, we suggest that when assessing the impacts of mountain roads on plant communities and postconstruction restoration efforts, more attention should be given to the loss of functional traits and older species rather than the spread of nonnative species or generalists, especially in the Hengduan Mountain region and similar ecosystems, where there are many endemic and relict species.

## Data availability statement

The original contributions presented in the study are included in the article/**Supplementary Material**. Further inquiries can be directed to the corresponding authors.

## Author contributions

HL: Conceptualization, Methodology, Investigation, Writing-original draft, Writing-review & editing. PL: Writing-review & editing, Funding acquisition. HY: Writing-review & editing. WX, CL, HJ, YC and YH: collected the trait data and conducted the field work. All authors contributed to the article and approved the submitted version.
