# Supplementary material for: Understanding the taxonomic homogenization of road-influenced plant assemblages in the Qionglai mountain range: A functional and phylogenetic perspective
Source: Front Plant Sci. 2023 Jan 9;13:1086185. doi: 10.3389/fpls.2022.1086185 (PMC9868858; doi:10.3389/fpls.2022.1086185)
Supplement: Supplementary file 1 [file DataSheet_1.docx]

# **Understanding the taxonomic homogenization of road-influenced plant assemblages in the Qionglai mountain range: A functional and phylogenetic perspective**

Honglin Li^a,b^, Peng Luo^a,b^^[[1]](#footnote-1)^, Hao Yang^a1^, Wenwen Xie^a,b^, Chuan Luo^a,b^, Honghong Jia^a,b^, Yue Cheng^a,b^, Yu Huang^a,b^

a. CAS Key Laboratory of Mountain Ecological Restoration and Bioresource Utilization & Ecological Restoration and Biodiversity Conservation Key Laboratory of Sichuan Province, Chengdu Institute of Biology, Chinese Academy of Sciences, Chengdu 610041, China

b. University of Chinese Academy of Sciences, Beijing 100049, China

## Supplementary information

## **Table S1** Functional traits of plant species and data sources (Flora of China, TRY, and measured in the laboratory).

| **Functional traits** | **Data name** | **Types of traits** | **Data sources** |
| --- | --- | --- | --- |
| Life form | Name data | Parasitic plant; Annual herbaceous; Biennial or annual herbaceous; Perennial herbaceous; Vine; Semi-shrub; Shrub; Tree | Flora of China |
| Leaf type | Name data | Fleshy leaf; Coriaceous leaf; Herbaceous leaf; Paper leaf; Membranous leaf | Flora of China |
| Reproductive mode | Name data | Sexual and asexual reproduction; Asexual reproduction | Flora of China |
| Seed dispersal models | Name data | Self-dispersal; Wind dispersal; Animal dispersal | TRY/Flora of China |
| Nitrogen-fixing | Binary data | Does not fix nitrogen; Nitrogen fixation | TRY/Flora of China |
| Root system | Name data | Tap root; Fibrous root | Flora of China |
| First month of bloom | Numerical data | The month in which the plant begins to bloom | Flora of China |
| Shade tolerance | Name data | Intolerant; Tolerant | TRY/Flora of China |
| Heliophile species | Name data | Yes; No | TRY/Flora of China |
| Special leaf area (SLA) (ratio of fresh leaf area to dry leaf mass) | Numerical data | / | Measured |
| Leaf thickness (L_th_) (mm) | Numerical data | / | Measured |
| Leaf dry matter mass (LDMC) (mg) | Numerical data | / | Measured |

## **Table S2** Phylogenetic signal in the 12 functional traits calculated for 978 species found according to Pagel’s λ metric. The significance is shown as (n.s.= non-significant; *** *P* < 0.001.)

| **Functional Trait** | **Types of traits** | **Pagel’s λ** | **P-value** |
| --- | --- | --- | --- |
| Life form | Annual herbaceous | 0.47 | *** |
|  | Biennial herbaceous | 0.27 | *** |
|  | Parasitic plant | 0.99 | *** |
|  | Perennial herbaceous | 0.87 | *** |
|  | Semi shrub | 0 | n.s. |
|  | Shrub | 0.95 | *** |
|  | Tree | 0.93 | *** |
|  | Vine | 0.99 | *** |
| Leaf type | Coriaceous leaf | 0.76 | *** |
|  | Fleshy leaf | 0.95 | *** |
|  | Herbaceous leaf | 0.55 | *** |
|  | Membranous leaf | 0.79 | *** |
|  | Paper leaf | 0.75 | *** |
| Root system | Root fibrous | 0.99 | *** |
|  | Root tap | 0.99 | *** |
| Reproductive mode | Asexual reproduction | 0.23 | n.s. |
|  | Sexual and asexual reproduction | 0.73 | *** |
| Seed dispersal models | Gravity dispersal | 0.99 | *** |
|  | Wind dispersal | 0.99 | *** |
|  | Animal dispersal | 0.98 | *** |
| Nitrogen fixation | | 0.99 | *** |
| Leaf thickness | | 0.88 | *** |
| Leaf dry matter content | | 0.99 | *** |
| Specific leaf area | | 0.99 | *** |
| First month of bloom | | 0.75 | *** |
| Heliophile species | | 0.13 | n.s. |
| Shade tolerance | | 0.07 | *** |

## **Table S3** Standardized direct, indirect and total effects of the soil variables on the three facets of plant diversity included in our structural equation models. Abbreviations: TBD, taxonomic beta diversity; FBD, functional beta diversity; bwPBD, basal-weighted beta diversity; twPBD, tip-weighted phylogenetic beta diversity.

| **Beta diversity** | | **Mountain roads** | **NO_3_^-^-N** | **SOM** | **pH** | **BK** | **FBD** | **PBD** | **MPD** |  |
| --- | --- | --- | --- | --- | --- | --- | --- | --- | --- | --- |
|  |  |  |  |  |  |  |  |  |  |  |
| **total effects** | | | | | | | | | |  |
| βtotal | FBD | -0.081 | 0.144 | -0.093 | 0.160 | 0.029 | - | - | - |  |
|  | bwPBD | **-0.510** | 0.037 | -0.034 | -0.019 | 0.129 | 0.312 | - | - |  |
|  | twPBD | -0.019 | 0.138 | -0.102 | 0.209 | 0.056 | **0.893** | - | - |  |
|  | TBD | -0.127 | -0.021 | -0.023 | 0.110 | 0.040 | **0.912** | 0.301 | -0.067 |  |
| βrepl | FBD | -0.043 | 0.146 | -0.079 | 0.141 | 0.014 | **-** | - | - |  |
|  | bwPBD | **-0.509** | 0.037 | -0.034 | -0.019 | 0.129 | 0.246 | - | - |  |
|  | twPBD | -0.086 | 0.119 | -0.043 | 0.185 | 0.011 | **0.755** | - | - |  |
|  | TBD | -0.048 | -0.210 | 0.056 | 0.046 | -0.019 | **0.653** | 0.206 | -0.209 |  |
| βrich | FBD | -0.031 | -0.054 | 0.010 | -0.026 | 0.014 | **-** | - | - |  |
|  | bwPBD | **-0.509** | 0.038 | -0.034 | -0.020 | 0.130 | -0.010 | - | - |  |
|  | twPBD | 0.112 | -0.020 | -0.040 | -0.022 | 0.006 | 0.461 | - | - |  |
|  | TBD | 0.078 | 0.370 | -0.096 | 0.067 | 0.041 | 0.471 | 0.113 | 0.270 |  |
| **direct effects** | | | | | | | | | |  |
| βtotal | FBD | -0.065 | 0.144 | -0.093 | 0.160 | 0.029 | - | - | - |  |
|  | bwPBD | -0.492 | -0.008 | -0.005 | -0.069 | 0.120 | 0.312 | - | - |  |
|  | twPBD | 0.061 | 0.009 | -0.019 | 0.066 | 0.030 | **0.893** | - | - |  |
|  | TBD | -0.096 | -0.044 | 0.044 | -0.021 | 0.020 | **0.664** | 0.301 | -0.067 |  |
| βrepl | FBD | -0.026 | 0.146 | -0.079 | 0.141 | 0.014 | **-** | - | - |  |
|  | bwPBD | **-0.505** | 0.001 | -0.015 | -0.054 | 0.126 | 0.246 | - | - |  |
|  | twPBD | -0.036 | 0.009 | 0.017 | 0.079 | 0.000 | **0.755** | - | - |  |
|  | TBD | -0.106 | -0.084 | 0.093 | -0.059 | 0.000 | **0.549** | 0.206 | -0.209 |  |
| βrich | FBD | -0.037 | -0.054 | 0.010 | -0.026 | 0.014 | **-** | - | - |  |
|  | bwPBD | **-0.512** | 0.037 | -0.034 | -0.020 | 0.130 | -0.010 | - | - |  |
|  | twPBD | 0.113 | 0.005 | -0.045 | -0.010 | 0.000 | 0.461 | - | - |  |
|  | TBD | 0.077 | 0.085 | -0.086 | 0.085 | 0.000 | 0.422 | 0.113 | 0.270 |  |
| **indirect effects** | | | | | | | | | |  |
| βtotal | FBD | -0.016 | 0 | 0 | 0 | 0 | - | - | - |  |
|  | bwPBD | -0.018 | 0.045 | -0.029 | 0.050 | 0.009 | - | - | - |  |
|  | twPBD | -0.080 | 0.129 | -0.083 | 0.143 | 0.026 | - | - | - |  |
|  | TBD | -0.031 | 0.023 | -0.067 | 0.131 | 0.020 | 0.248 | - | - |  |
| βrepl | FBD | -0.017 | 0 | 0 | 0 | 0 | - | - | - |  |
|  | bwPBD | -0.004 | 0.036 | -0.019 | 0.035 | 0.003 | - | - | - |  |
|  | twPBD | -0.050 | 0.110 | -0.060 | 0.106 | 0.011 | - | - | - |  |
|  | TBD | 0.058 | -0.126 | -0.037 | 0.105 | -0.019 | 0.104 | - | - |  |
| βrich | FBD | 0.006 | 0 | 0 | 0 | 0 | - | - | - |  |
|  | MPD | 0.0031 | 0.0005 | -0.0001 | 0.0003 | -0.0001 | - | - | - |  |
|  | PBD | -0.001 | -0.025 | 0.005 | -0.012 | 0.006 | - | - | - |  |
|  | TBD | 0.001 | 0.285 | -0.010 | -0.018 | 0.041 | 0.049 | - | - |  |

| 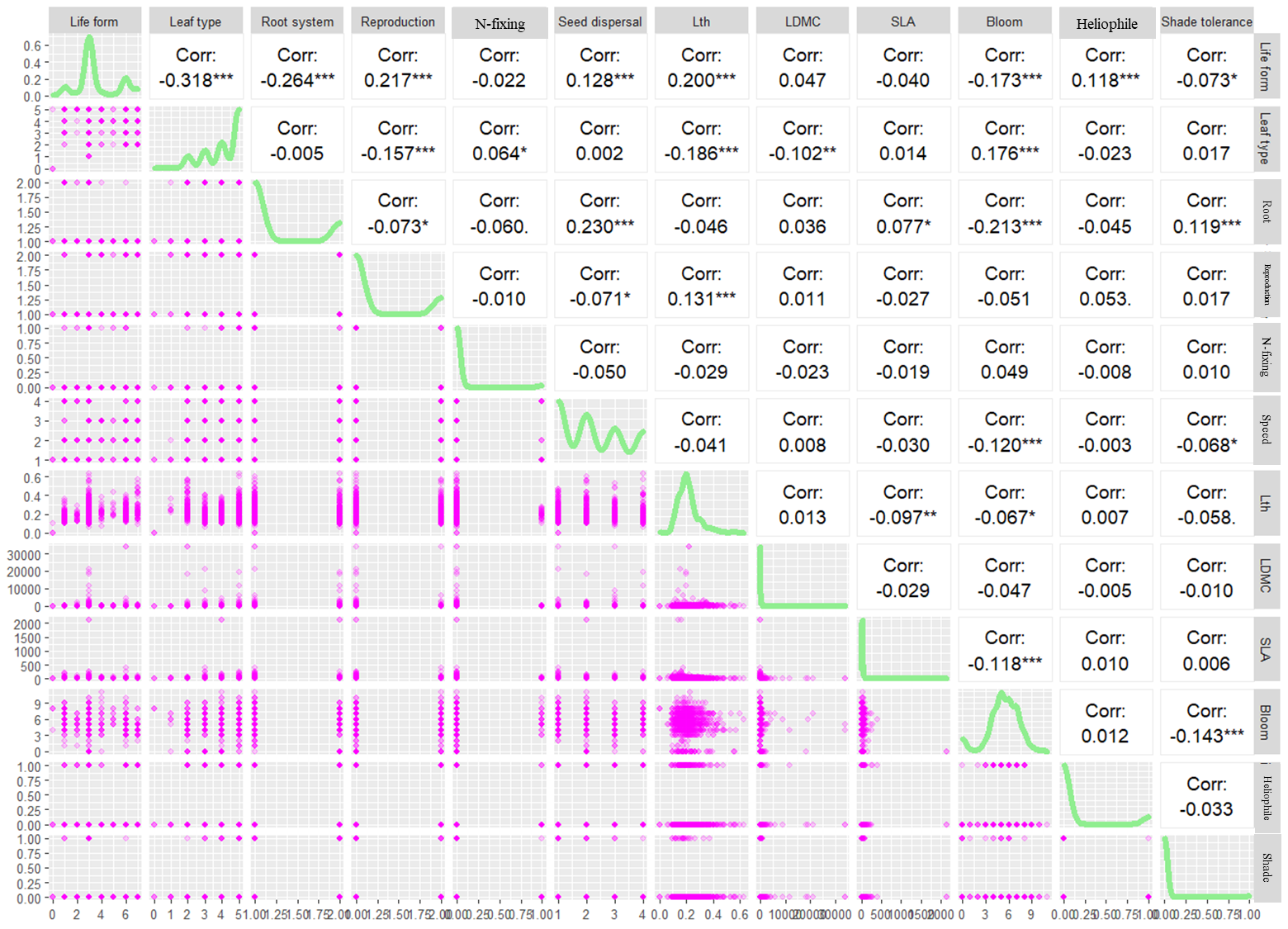 |
| --- |

## **Figure S1** The Pearson’s correlation within the 12 plant functional traits.

| 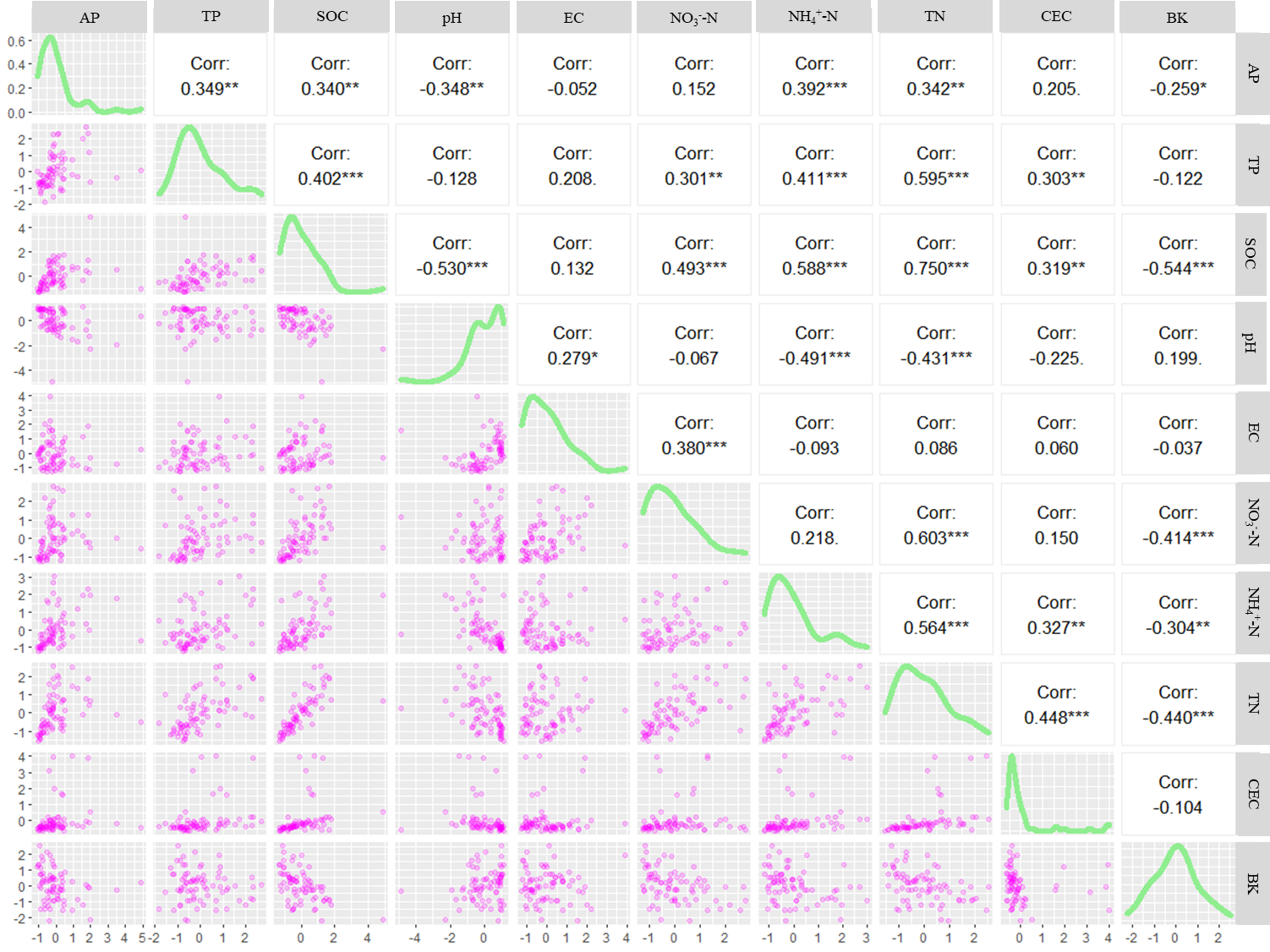 |
| --- |

## **Figure S2** The Pearson’s correlation within the 10 soil factors.

| 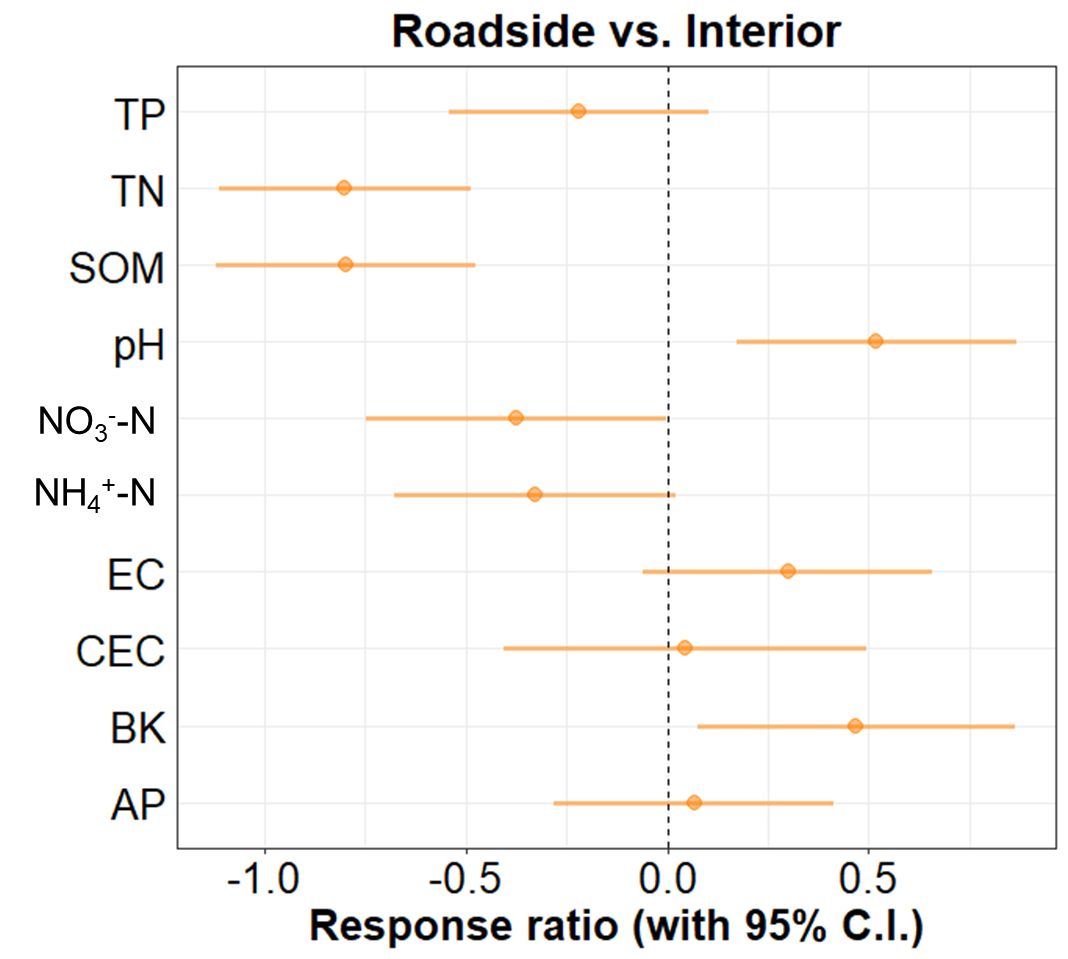 |
| --- |

## **Figure S3** Linear mixed modeling of soil factor as a function of plot category separately.

| 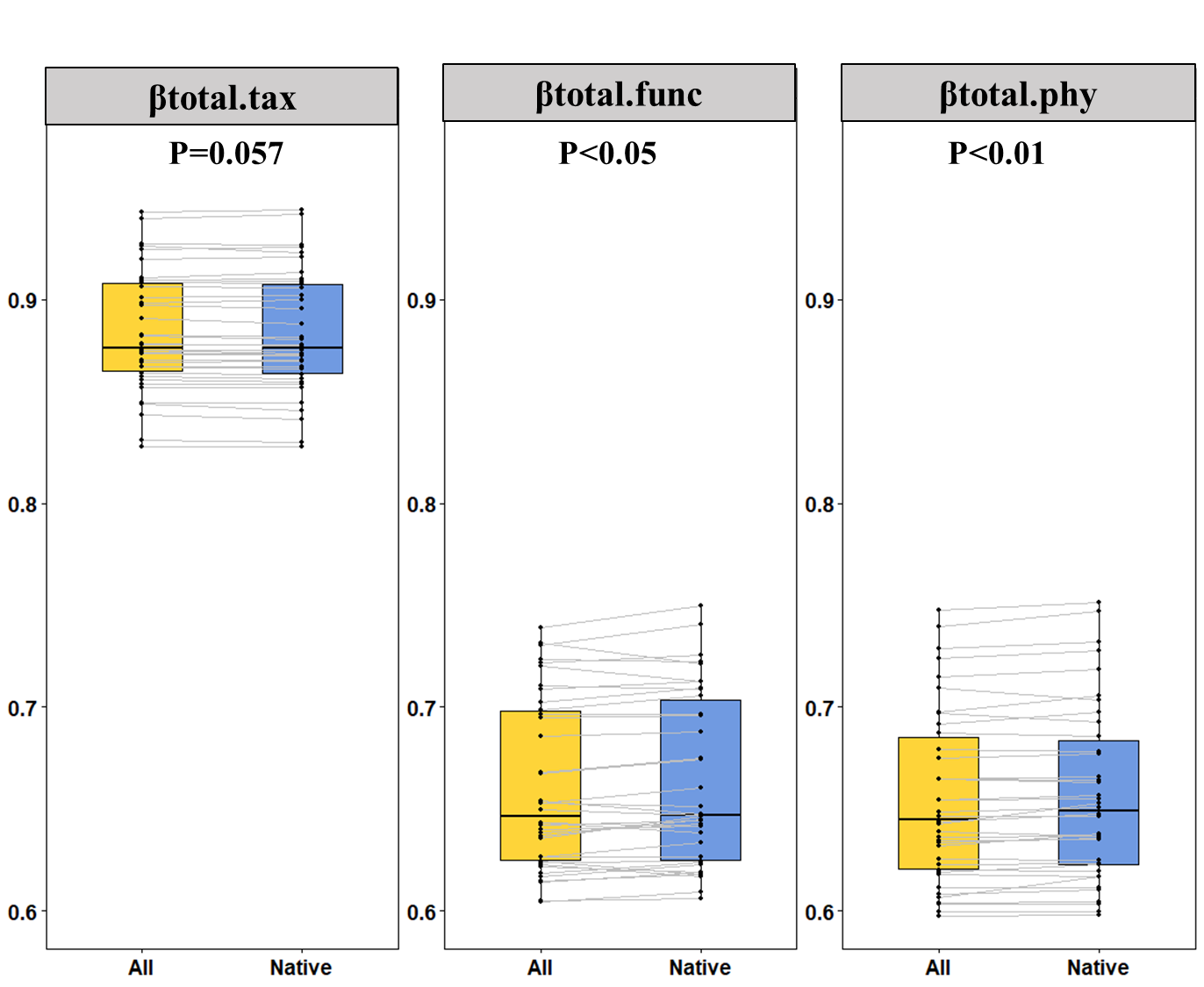 |
| --- |
| 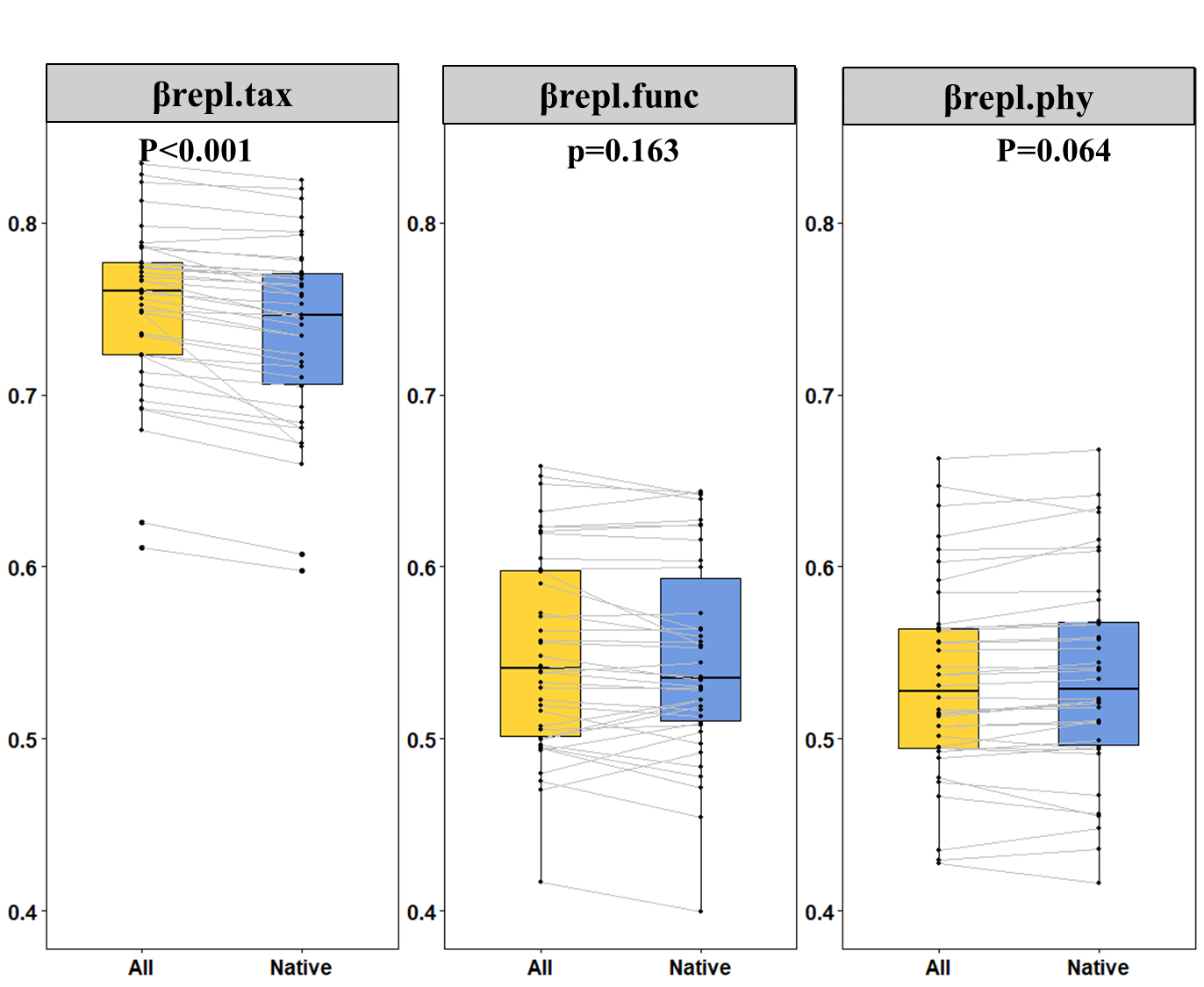 |
| 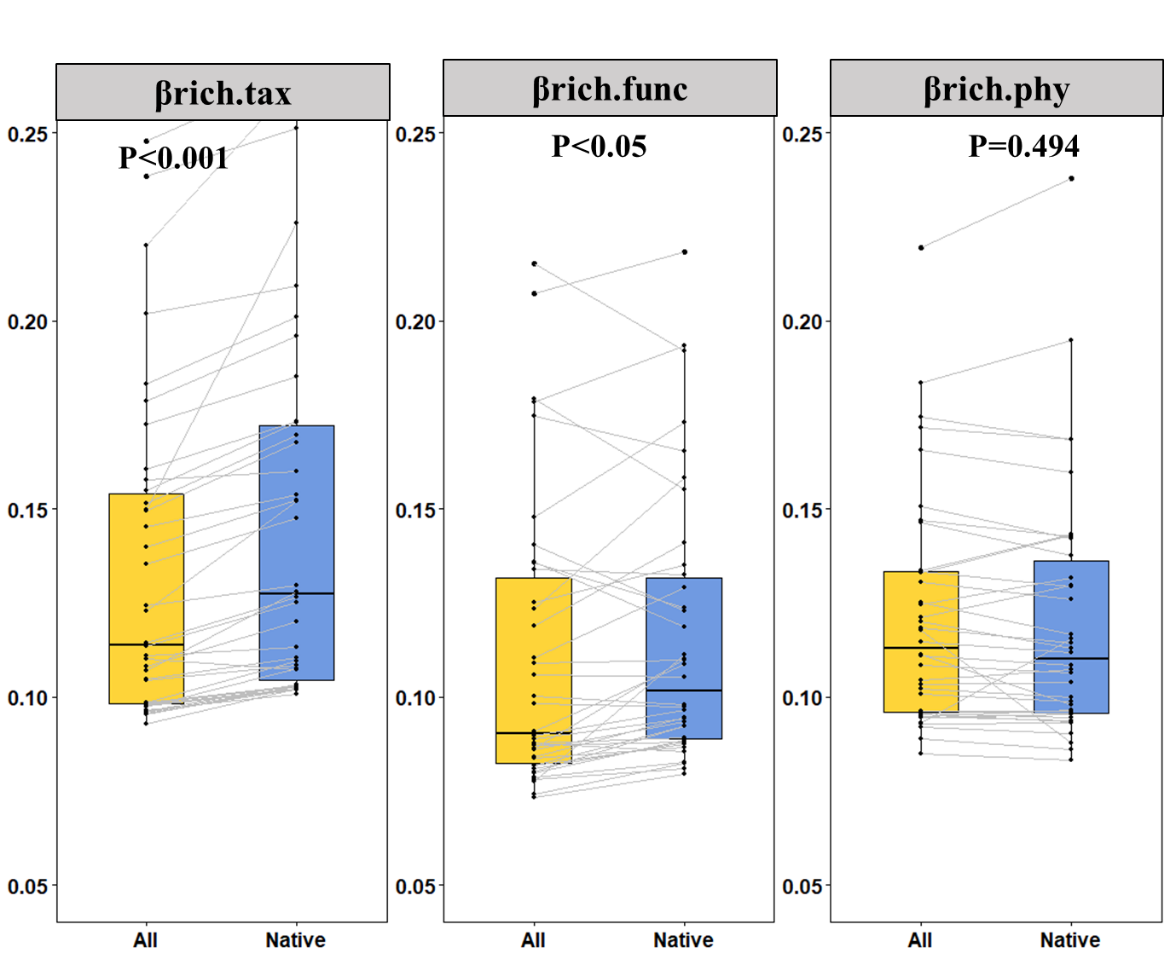 |
| 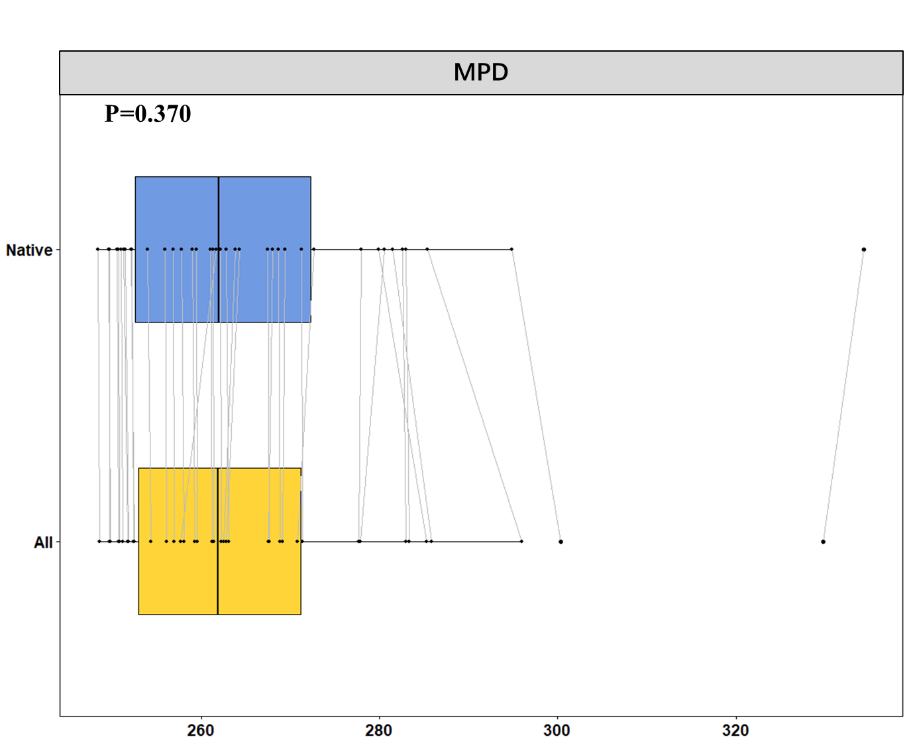 |

## **Figure S4** Differences between all species and native species were tested with linear mixed models (n=38). The points represent the mean beta diversity of each plot, and the lines connect the beta diversity of all species and native species in the roadside plot. The *P* value represents the result of the linear mixed effects model.

| 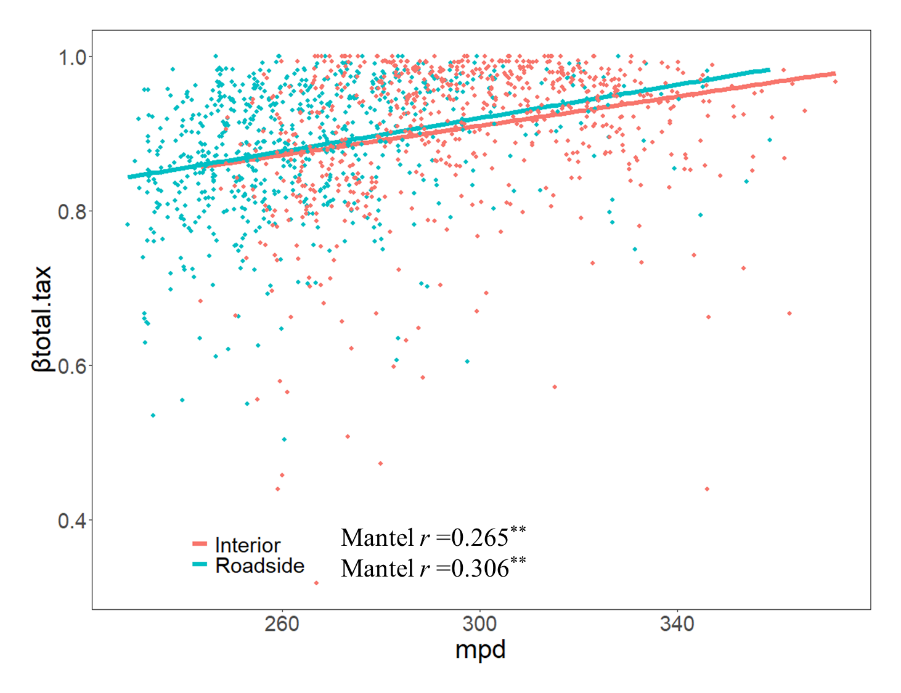 |
| --- |
| 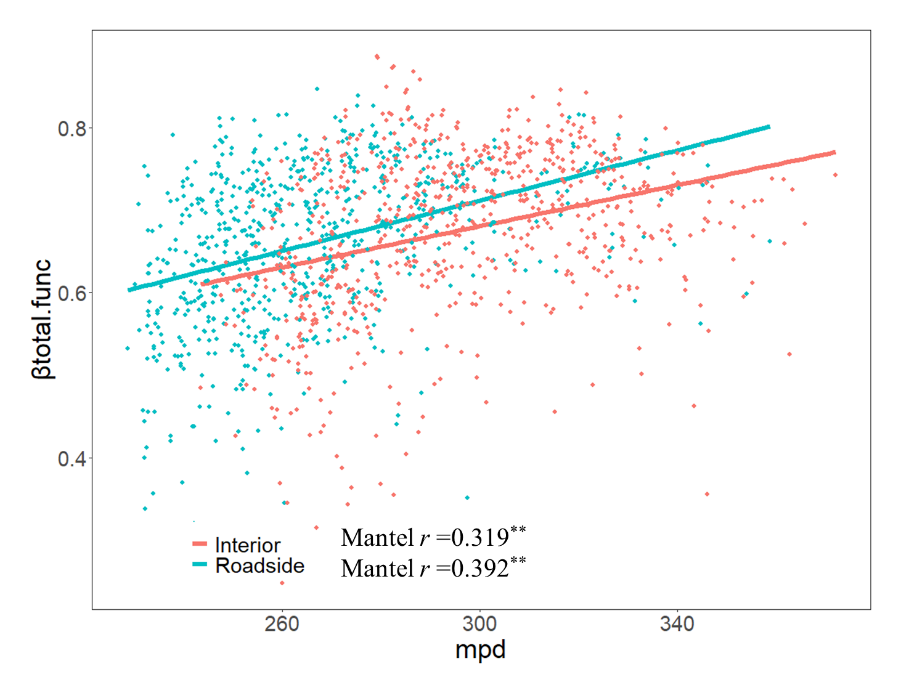 |
| 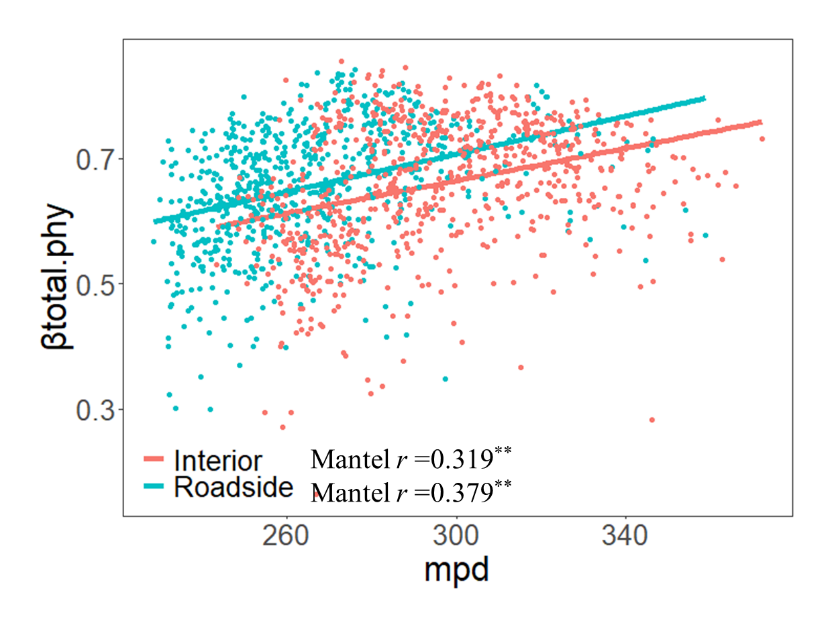 |
| 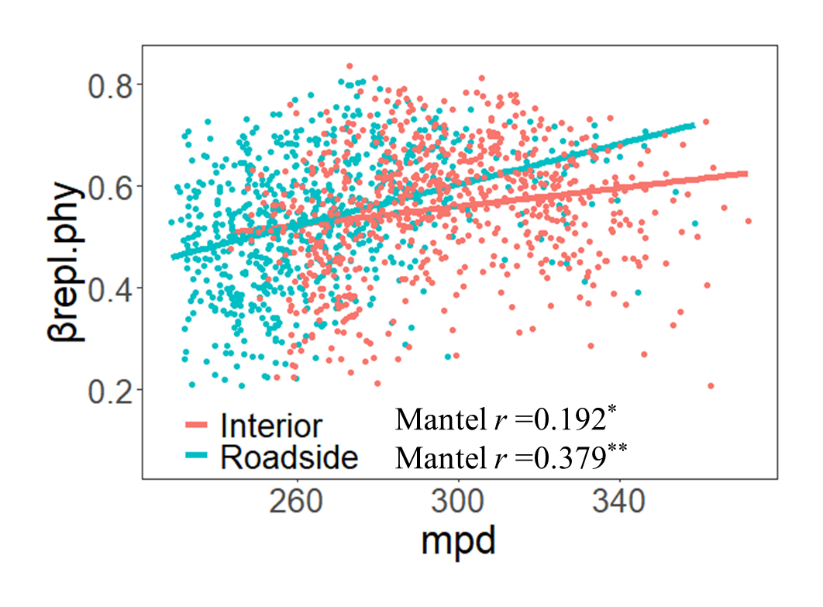 |

## **Figure S5** Correlations between mpd and βtotal.tax, βtotal.func, and βtotal.phy and βrepl.phy. ** *P* <0 .01, * *P* <0 .05.

| **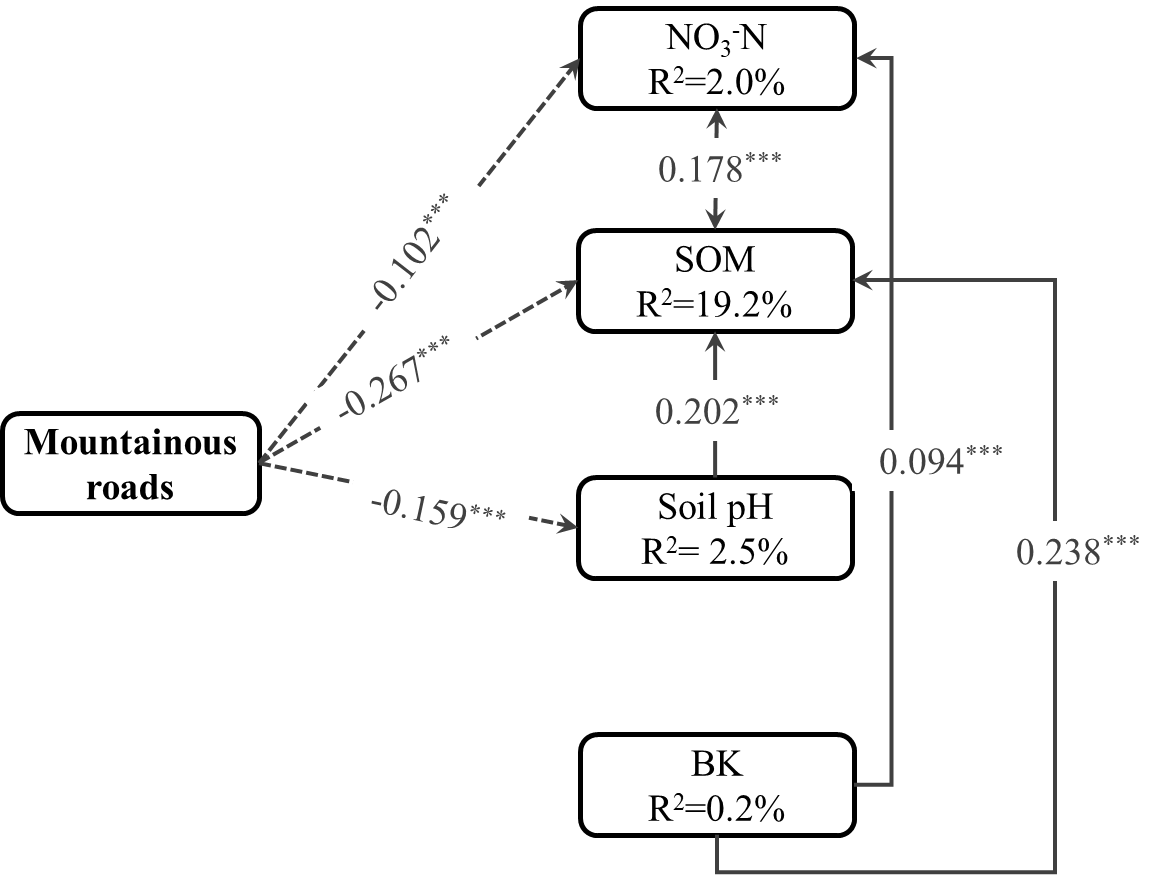** |
| --- |

## **Figure S6** Representation of direct and indirect influences of mountain roads on edaphic variables. Boxes indicate measured variables and arrows indicate relationships among variables. Single-headed arrows represent a one way directed relationship. Solid and broken arrows represent positive relationships and negative effects, respectively. Standardized path coefficients were given on each path. Significance levels are as follows: ****P* < 0.001.

1. Corresponding authors.

   E-mail addresses: luopeng@cib.ac.cn (Peng Luo), yanghao@cib.ac.cn (Hao Yang). [↑](#footnote-ref-1)
